# Can radiomics-based innovations improve the diagnosis of kidney fibrosis in diabetic nephropathy?

**DOI:** 10.1093/ckj/sfag050

**Published:** 2026-02-14

**Authors:** Yan Yao, Yan Ma, Yujie Jin, Mengru Wang, Chunchen Ni, Shujuan Shang, Chunyan Xing, Zhanyan Zhang, Kang Xie, JinHao Liu, Lizhuo Wang, Shiqiang Liu, Jialin Gao

**Affiliations:** Department of Endocrinology and Genetic Metabolism, The First Affiliated Hospital of Wannan Medical University (Yijishan Hospital of Wannan Medical University), Wuhu, Anhui, China; Institute of Endocrine and Metabolic Diseases, The First Affiliated Hospital of Wannan Medical University (Yijishan Hospital of Wannan Medical University), Wuhu, Anhui, China; Anhui Province Key Laboratory of Basic d Research anTransformation of Age-related Diseases, Wannan Medical University, Wuhu, Anhui, China; Department of Endocrinology and Genetic Metabolism, The First Affiliated Hospital of Wannan Medical University (Yijishan Hospital of Wannan Medical University), Wuhu, Anhui, China; Department of Endocrinology and Genetic Metabolism, The First Affiliated Hospital of Wannan Medical University (Yijishan Hospital of Wannan Medical University), Wuhu, Anhui, China; Department of Endocrinology and Genetic Metabolism, The First Affiliated Hospital of Wannan Medical University (Yijishan Hospital of Wannan Medical University), Wuhu, Anhui, China; Institute of Endocrine and Metabolic Diseases, The First Affiliated Hospital of Wannan Medical University (Yijishan Hospital of Wannan Medical University), Wuhu, Anhui, China; Anhui Province Key Laboratory of Basic d Research anTransformation of Age-related Diseases, Wannan Medical University, Wuhu, Anhui, China; Department of Endocrinology and Genetic Metabolism, The First Affiliated Hospital of Wannan Medical University (Yijishan Hospital of Wannan Medical University), Wuhu, Anhui, China; Institute of Endocrine and Metabolic Diseases, The First Affiliated Hospital of Wannan Medical University (Yijishan Hospital of Wannan Medical University), Wuhu, Anhui, China; Anhui Province Key Laboratory of Basic d Research anTransformation of Age-related Diseases, Wannan Medical University, Wuhu, Anhui, China; Department of Endocrinology and Genetic Metabolism, The First Affiliated Hospital of Wannan Medical University (Yijishan Hospital of Wannan Medical University), Wuhu, Anhui, China; Institute of Endocrine and Metabolic Diseases, The First Affiliated Hospital of Wannan Medical University (Yijishan Hospital of Wannan Medical University), Wuhu, Anhui, China; Anhui Province Key Laboratory of Basic d Research anTransformation of Age-related Diseases, Wannan Medical University, Wuhu, Anhui, China; Department of Endocrinology and Genetic Metabolism, The First Affiliated Hospital of Wannan Medical University (Yijishan Hospital of Wannan Medical University), Wuhu, Anhui, China; Institute of Endocrine and Metabolic Diseases, The First Affiliated Hospital of Wannan Medical University (Yijishan Hospital of Wannan Medical University), Wuhu, Anhui, China; Department of Endocrinology and Genetic Metabolism, The First Affiliated Hospital of Wannan Medical University (Yijishan Hospital of Wannan Medical University), Wuhu, Anhui, China; Institute of Endocrine and Metabolic Diseases, The First Affiliated Hospital of Wannan Medical University (Yijishan Hospital of Wannan Medical University), Wuhu, Anhui, China; Anhui Province Key Laboratory of Basic d Research anTransformation of Age-related Diseases, Wannan Medical University, Wuhu, Anhui, China; Department of Endocrinology and Genetic Metabolism, The First Affiliated Hospital of Wannan Medical University (Yijishan Hospital of Wannan Medical University), Wuhu, Anhui, China; Institute of Endocrine and Metabolic Diseases, The First Affiliated Hospital of Wannan Medical University (Yijishan Hospital of Wannan Medical University), Wuhu, Anhui, China; Anhui Province Key Laboratory of Basic d Research anTransformation of Age-related Diseases, Wannan Medical University, Wuhu, Anhui, China; Department of Endocrinology and Genetic Metabolism, The First Affiliated Hospital of Wannan Medical University (Yijishan Hospital of Wannan Medical University), Wuhu, Anhui, China; Institute of Endocrine and Metabolic Diseases, The First Affiliated Hospital of Wannan Medical University (Yijishan Hospital of Wannan Medical University), Wuhu, Anhui, China; Anhui Province Key Laboratory of Basic d Research anTransformation of Age-related Diseases, Wannan Medical University, Wuhu, Anhui, China; Anhui Province Key Laboratory of Basic d Research anTransformation of Age-related Diseases, Wannan Medical University, Wuhu, Anhui, China; Department of Biochemistry and Molecular Biology, Wannan Medical University, Wuhu, Anhui, China; Department of Endocrinology and Genetic Metabolism, The First Affiliated Hospital of Wannan Medical University (Yijishan Hospital of Wannan Medical University), Wuhu, Anhui, China; Institute of Endocrine and Metabolic Diseases, The First Affiliated Hospital of Wannan Medical University (Yijishan Hospital of Wannan Medical University), Wuhu, Anhui, China; Anhui Province Key Laboratory of Basic d Research anTransformation of Age-related Diseases, Wannan Medical University, Wuhu, Anhui, China; Department of Endocrinology and Genetic Metabolism, The First Affiliated Hospital of Wannan Medical University (Yijishan Hospital of Wannan Medical University), Wuhu, Anhui, China; Institute of Endocrine and Metabolic Diseases, The First Affiliated Hospital of Wannan Medical University (Yijishan Hospital of Wannan Medical University), Wuhu, Anhui, China; Anhui Province Key Laboratory of Basic d Research anTransformation of Age-related Diseases, Wannan Medical University, Wuhu, Anhui, China

**Keywords:** diabetic kidney disease, fibrosis, personalized treatment, radiomics

## Abstract

Radiomics is a promising quantitative imaging technique that extracts and analyzes high-throughput features from medical images, providing detailed structural and functional information. It has gained significant attention in diabetic kidney disease (DKD) research, particularly in assessing renal fibrosis and predicting treatment outcomes. Radiomics offers a novel approach for accurate DKD diagnosis and holds potential for personalized treatment strategies. When combined with artificial intelligence and machine learning, it can create predictive models that improve clinical decision-making. Integrating radiomics with genomics and metabolomics further enhances understanding of disease mechanisms and facilitates biomarker discovery. Despite its potential, challenges such as lack of standardization, complex feature selection, limited model interpretability and inadequate clinical validation remain. Future advancements in imaging technologies, more efficient algorithms and large-scale clinical studies are expected to establish radiomics as a critical tool in precision medicine for DKD, enabling more accurate and personalized non-invasive diagnostics and therapies in nephrology.

## GRAPHICAL ABSTRACT

**GRAPHICAL ABSTRACT:**
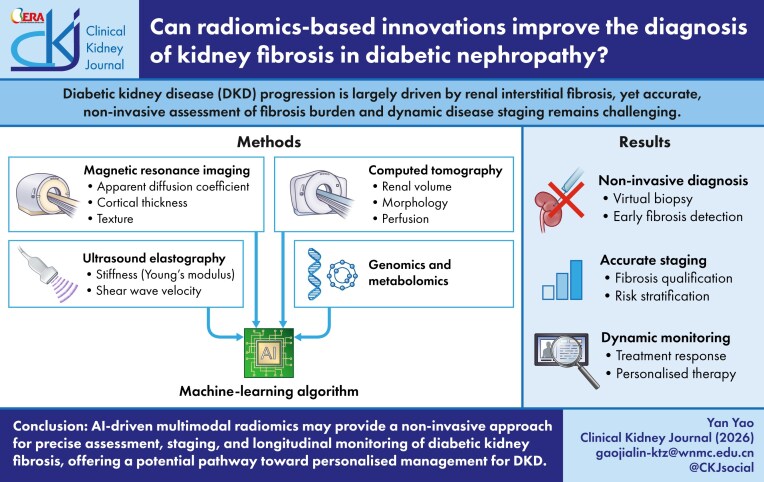

## INTRODUCTION

Diabetic kidney disease (DKD) is one of the most common chronic complications among individuals with diabetes and represents a leading cause of end-stage renal disease (ESRD) worldwide [1]. Its pathological hallmarks include glomerulosclerosis, tubulointerstitial fibrosis and persistent inflammation, which collectively contribute to the progressive loss of renal function [2]. Although current clinical diagnosis primarily depends on conventional biomarkers such as glomerular filtration rate (GFR) and urinary protein levels [3], these indicators mainly reflect the functional decline of the kidney rather than early structural changes. Among the pathological features, renal fibrosis plays a central role in DKD progression. The extent of fibrosis not only correlates with the rate of renal function deterioration but also serves as a key determinant of long-term clinical outcomes [4]. Traditional methods for assessing renal fibrosis, such as kidney biopsy, offer precise histological information but are invasive and unsuitable for repeated or dynamic monitoring. This limitation underscores the urgent need for non-invasive, reliable tools to evaluate and track fibrosis progression over time.

Radiomics provides a novel, non-invasive approach for quantitatively assessing renal fibrosis. By extracting high-throughput features from medical images, radiomics enables the detection of fibrosis-related patterns and facilitates real-time, longitudinal evaluation of disease progression. Recent advances in artificial intelligence (AI) and machine learning (ML) have further enhanced the ability of radiomics to identify subtle structural changes that are not discernible through conventional imaging.

This study aims to provide a comprehensive review of current progress in the application of radiomics in DKD, with a particular focus on fibrosis evaluation and prediction of treatment outcomes. By integrating multimodal imaging data with advanced computational algorithms, this review highlights the potential of radiomics as a tool for precision medicine in DKD. Furthermore, we discuss key challenges, including data standardization, model interpretability and clinical validation, as well as potential solutions. The innovative aspect of this work lies in the convergence of radiomic features with ML techniques to improve diagnostic accuracy, guide personalized therapies and support early intervention strategies. For this review, a comprehensive literature search was conducted in PubMed, Scopus and Web of Science databases, using the keywords “radiomics,” “diabetic kidney disease,” “fibrosis,” “MRI,” “CT,” “shear wave elastography” and “artificial intelligence.” Studies published from 2021 to 2024 were included, focusing on human subjects and clinical applications. Exclusion criteria included animal studies, non-English publications and articles not related to DKD. Ultimately, this review seeks to promote the clinical translation of radiomics in the management of DKD and contributes to the development of more effective, individualized treatment paradigms.

## MRI-BASED RENAL FIBROSIS EVALUATION

Magnetic resonance imaging (MRI) is a widely used imaging modality for assessing renal fibrosis, owing to its excellent soft tissue contrast and non-invasive nature [5]. Compared with conventional diagnostic techniques, MRI enables detailed visualization of renal structure and provides quantitative parameters relevant to fibrosis. In recent years, the integration of MRI with radiomics has significantly advanced the field of fibrosis evaluation in DKD.

One important MRI-derived parameter is renal cortical thickness, which serves as an indicator of disease progression in DKD. Cortical thinning is often observed in the early stages of DKD and is associated with worsening renal function. Regular assessment of cortical thickness enables clinicians to monitor structural changes and treatment responses over time. MRI texture analysis allows for the extraction of radiomic features that reflect microscopic structural alterations in renal tissue. In a retrospective study, Yu *et al*. analyzed MRI data from 44 healthy volunteers and 40 patients with stage III type 2 diabetic nephropathy and microalbuminuria. Using 1.5T conventional MRI and diffusion-weighted imaging (DWI), the study applied LASSO logistic regression to identify 10 key texture features. These features were used to construct a diagnostic model for diabetic nephropathy, which achieved excellent performance: an area under the curve (AUC) of 0.98, specificity of 0.97, accuracy of 0.93 and sensitivity exceeding 92% [6]. The extracted features—including Gray Level Co-occurrence Matrix (GLCM) and Gray Level Run Length Matrix (GLRLM)—demonstrated strong potential for early diabetic nephropathy detection without the need for contrast agents.

In addition to conventional sequences, contrast-enhanced MRI techniques offer valuable functional insights. Dynamic contrast-enhanced MRI (DCE-MRI) enables the evaluation of GFR, renal blood perfusion and vascular integrity. Enhancement patterns on DCE-MRI can detect vascular abnormalities such as stenosis, dilation and occlusion, which are critical for assessing DKD progression.

DWI and diffusion tensor imagingrepresent additional non-invasive MRI modalities widely used to assess renal fibrosis [7, 8]. These techniques measure water molecule diffusion within tissues, which inversely correlates with fibrotic severity. A study by Hiroya Kitsunai *et al [9]* . compared apparent diffusion coefficient (ADC) values among type 2 diabetes patients, hypertensive patients and healthy controls. ADC values were significantly lower in diabetic patients and positively correlated with estimated GFR (eGFR), while showing inverse correlations with serum creatinine, cystatin C and urine albumin–creatinine ratio. The study concluded that cortical ADC values may serve as quantitative indicators of tubulointerstitial fibrosis and renal injury progression. Lower ADC values were strongly associated with more severe fibrosis.

Moreover, magnetic resonance elastography (MRE) is an emerging MRI-based technique capable of assessing tissue stiffness—a direct surrogate of fibrosis [10]. Research by Michael JK and colleagues [11] demonstrated that medullary stiffness was significantly increased in kidneys affected by renal artery stenosis compared with healthy kidneys (12.7 ± 0.41 kPa vs 10.7 ± 0.18 kPa; *P* = .004). MRE-derived stiffness measurements showed strong correlations with histopathological fibrosis scores, indicating its potential value in non-invasive fibrosis quantification and longitudinal monitoring.

Radiomics-based analysis of MRI features—such as texture, shape and signal intensity—enhances the precision of renal fibrosis evaluation. These features often correlate closely with biopsy-derived fibrosis scores and may serve as reliable alternatives for fibrosis quantification. MRI provides superior tissue contrast and multiparametric data, but its clinical use is limited by high cost, accessibility issues and contraindications in patients with implants or claustrophobia. Its sensitivity to subtle fibrotic changes is promising but requires standardization across scanners and centers. MRI-based radiomics could be used as a second-line evaluation tool after routine ultrasound (US) or lab-based markers suggest progression, offering detailed fibrosis mapping to guide treatment stratification.

## ULTRASOUND ELASTOGRAPHY (SWE)-BASED RENAL FIBROSIS EVALUATION

Shear wave elastography (SWE) is a non-invasive imaging technique that quantitatively evaluates tissue elasticity and has emerged as a promising tool for assessing renal fibrosis in DKD [12, 13]. In recent years, the application of SWE technology in DKD has made significant progress. A study by Krehl *et al*. indicated that regions of renal fibrosis exhibit stiffer tissue with higher elasticity coefficients, suggesting more severe fibrosis [14]. SWE radiomic analysis can accurately assess the degree of fibrosis by extracting elasticity features, such as shear wave speed and tissue stiffness [15, 16].

SWE functions by emitting acoustic radiation force impulses into tissues to generate shear waves and subsequently measuring their propagation speed. The velocity of shear wave propagation is positively correlated with tissue stiffness—stiffer tissues produce faster shear wave speeds [17]. Using these measurements, the Young’s modulus (YM) can be calculated to quantify tissue elasticity, where a higher YM value indicates increased fibrosis severity [18]. Recent studies have demonstrated the clinical utility of SWE in detecting and monitoring renal fibrosis. In a prospective study involving 93 healthy controls and 108 patients with chronic kidney disease, SWE was used to assess renal cortical stiffness using YM as a key parameter. The analysis revealed that a YM threshold of 4.43 kPa could effectively distinguish healthy from fibrotic renal tissue, with a sensitivity of 92.6%, specificity of 80.6% and an area under the receiver operating characteristic curve of 0.92 [15, 19]. Furthermore, Pearson correlation analysis showed that SWE-derived stiffness parameters were significantly associated with conventional renal function indicators, such as serum creatinine, eGFR and serum urea levels.

Beyond diagnosis, SWE also provides valuable tools for treatment monitoring. When combined with radiomic techniques, SWE enables dynamic and non-invasive tracking of fibrosis progression and therapeutic responses in DKD. For instance, changes in renal stiffness following antifibrotic therapy can be captured in real time, offering clinicians feedback on treatment efficacy and guiding personalized intervention strategies. Given its widespread availability, low cost and non-invasive nature, SWE is particularly well-suited for routine follow-up and longitudinal fibrosis monitoring in DKD patients. Its integration with radiomics represents a powerful strategy for enhancing early diagnosis, precision monitoring and individualized care in diabetic nephropathy.

While MRI offers excellent soft tissue contrast, it is often limited by accessibility and patient compatibility, especially in those with implantable devices.In contrast, while SWE is non-invasive and inexpensive, it suffers from operator dependence and technical variability, and may be less reliable in certain clinical settings. Differences in probe pressure, patient body habitus and region-of-interest selection can lead to inconsistent results across centers. SWE can be deployed in outpatient clinics as a first-line fibrosis screening tool, and is especially suitable for longitudinal monitoring. Coupling SWE-derived stiffness indices with radiomic features may enhance early DKD stratification.

## CT-BASED FIBROSIS EVALUATION

Diabetes influences renal morphology, particularly kidney volume, which has been shown to correlate with markers of renal function—such as GFR—and glycemic control indicators like hemoglobin A1c [20]. A reduction in kidney volume may be indicative of progressive diabetic kidney fibrosis [21]. While conventional computed tomography (CT) and contrast-enhanced CT (CE-CT) do not directly visualize fibrotic tissue, they can detect morphological and perfusion-related changes that indirectly reflect fibrotic progression. For instance, in DKD, CT imaging often reveals renal atrophy and pelvicalyceal system dilatation. CE-CT can assess renal vascular perfusion through the analysis of enhancement patterns and time–density curves in renal arteries, veins and the collecting system, providing surrogate indicators of impaired blood flow . In a preclinical study, Margarita Braunagel et al. [22] induced warm ischemia/reperfusion injury in wild-type mice for 45 or 60 min, followed by CE-CT imaging on Days 2, 7 and 18 post-injury. The study quantitatively assessed renal blood flow, showing significantly reduced perfusion on Days 2 and 7, with partial recovery by Day 18—particularly in the less severely injured group. These findings support the use of CE-CT to detect fibrosis-associated perfusion deficits and their temporal evolution.

Despite these insights, CT-based methods have inherent limitations in the direct assessment of fibrosis due to low sensitivity to early extracellular matrix remodeling and radiation exposure concerns. In clinical practice, CT is often used as part of a multimodal imaging approach, in combination with MRI, US elastography and emerging omics data—including genomics, transcriptomics and metabolomics—to achieve a more comprehensive evaluation of DKD-related fibrosis.

Recent advances in radiomics, particularly when integrated with AI and ML techniques, have expanded the potential of CT imaging in fibrosis assessment [23]. Deep learning (DL) algorithms can automatically extract high-dimensional imaging features, uncover complex data patterns and enhance the predictive performance of radiomics models. For example, Jimenez-Mesa *et al*. reviewed the application of ML and DL in nuclear imaging modalities such as single photon emission CT (SPECT) and positron emission tomography (PET), demonstrating their value in image acquisition optimization, biomarker identification, and the development of diagnostic and prognostic tools [24]. In another study, Christie *et al*. applied radiomics feature extraction and random survival forest modeling to CT and FDG PET-CT images of 135 patients. Their model effectively predicted recurrence or progression timelines and distinguished between high- and low-risk patients in both training and testing cohorts [25].

Furthermore, the integration of DL with radiomic pipelines in CT imaging enables automated identification of fibrosis-associated features. This reduces operator-dependent variability and improves the reproducibility and accuracy of fibrosis quantification [26, 27]. CT imaging provides morphological insights but exposes patients to radiation and lacks sensitivity for early extracellular matrix remodeling. Contrast use may be risky in patients with impaired kidney function. CT radiomics could be considered in multimodal protocols for patients already undergoing abdominal CT for other indications, extracting additional fibrosis data without requiring separate imaging sessions.

## MULTIMODAL IMAGING-BASED FIBROSIS EVALUATION

**Figure 1:**
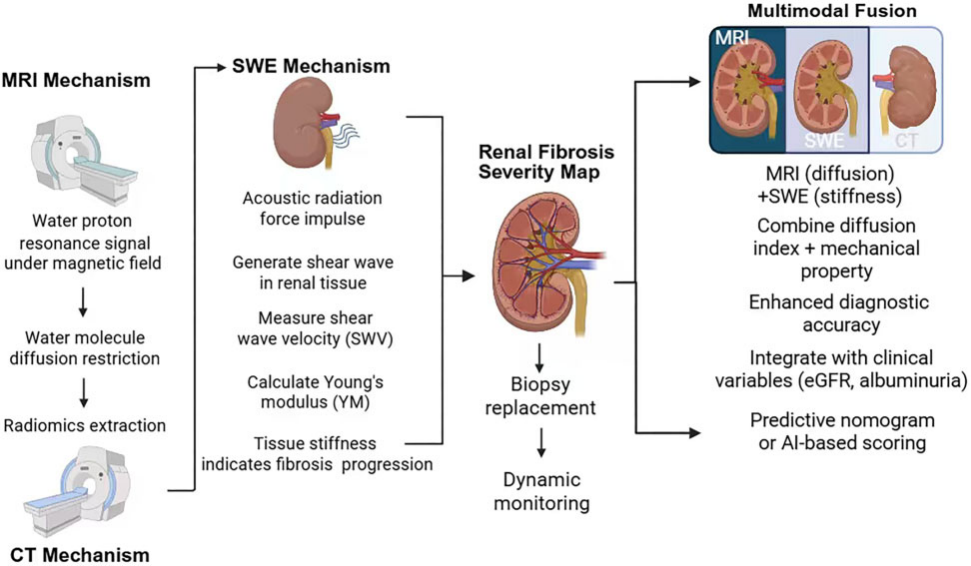
Non-invasive imaging-based mechanisms for renal fibrosis assessment. MRI, SWE and CT synergistically construct renal fibrosis severity maps by assessing diffusion, stiffness and structural features. Multimodal fusion enhances diagnostic accuracy and enables biopsy-free dynamic monitoring. Integration with clinical parameters (eGFR, albuminuria) allows predictive modeling through nomograms or AI scoring, offering a robust tool for fibrosis quantification and therapeutic evaluation.（Figure created with BioRender.com）

Single imaging modalities each have inherent limitations in evaluating renal fibrosis. In contrast, multimodal radiomics, which integrates data from various imaging techniques, offers a more comprehensive and multidimensional approach to fibrosis assessment [28]. By combining structural, functional, and mechanical information from different imaging platforms, multimodal radiomics enhances both the sensitivity and specificity of fibrosis detection (Fig. 1).

US elastography and MRI provide complementary diagnostic insights. US elastography enables non-invasive evaluation of renal tissue stiffness, while MRI offers high-resolution anatomical imaging and detailed microstructural assessment. In a study by Meyers *et al*., the combined use of US and MRI was shown to improve diagnostic performance in pediatric soft tissue lesion evaluation, demonstrating the utility of dual-modality imaging in clinical decision-making [29]. Multimodal radiomics integrating MRI DWI and SWE has shown superior accuracy in renal fibrosis staging compared with single-modality analysis. By simultaneously capturing tissue diffusion characteristics and elasticity parameters, this combination yields a stronger correlation with histopathological fibrosis scores. In a representative study, Xin-Yue Ge *et al. [19]* stratified cortical tubulointerstitial fibrosis (interstitial fibrosis and tubular atrophy, IFTA) into mild, moderate and severe stages. Two classification schemes were tested: (i) distinguishing mild from moderate-to-severe IFTA, and (ii) distinguishing mild-to-moderate from severe IFTA. Multivariable logistic regression integrating clinical features (e.g. albumin, eGFR, age) and radiomic features was used to construct a predictive nomogram. Decision curve analysis demonstrated that this integrated model outperformed both clinical and radiomic models alone (*P* < .05, DeLong test), highlighting the value of multimodal fusion.

Beyond MRI and US, texture features from CT have also been incorporated into multimodal radiomics pipelines. These features, including markers of glomerulosclerosis and cortical atrophy, can complement DWI- and signal intensity–based MRI features to improve fibrosis characterization. In a study by Auer *et al*., multimodal data from US, CT and MRI were fused to assess renal lesions in 28 patients. The results confirmed that such fusion improved diagnostic accuracy, particularly for lesions that were difficult to localize using grayscale US alone [30].

While multimodal fusion can substantially enhance diagnostic accuracy, several challenges remain, including increased cost, logistical complexity and the absence of universally accepted fusion protocols. A major barrier is data harmonization, as inconsistencies in image acquisition, reconstruction parameters and feature extraction pipelines can significantly limit reproducibility. To address these concerns, several international standardization efforts are underway. For example, the Image Biomarker Standardisation Initiative aims to establish consensus guidelines for image acquisition, preprocessing and radiomic feature computation, thereby ensuring methodological consistency across studies [31]. In parallel, professional societies such as the European Society of Radiology and the American College of Radiology are actively developing guidelines to promote the standardized use of imaging biomarkers in research and clinical practice [32]. These initiatives collectively play a crucial role in improving the reproducibility of radiomics models and facilitating their translation into clinical applications across diverse patient populations.

In the context of DKD, the importance of standardization is further amplified due to the heterogeneity of imaging modalities, patient demographics and disease stages. Implementing standardized acquisition and processing workflows can markedly enhance the reliability of radiomics-derived assessments of renal fibrosis. Moreover, harmonized protocols enable the design of large-scale, multicenter studies and streamline future regulatory approval pathways—both essential steps toward the widespread clinical adoption of radiomics-based tools for DKD evaluation [33–35]. By embracing these standardization frameworks, the clinical utility of radiomics can be significantly strengthened, ultimately supporting more accurate, reproducible and generalizable imaging biomarkers for DKD.

Multimodal pipelines combining MRI, SWE and CT with omics data may form the backbone of future DKD precision diagnostics, offering comprehensive fibrosis staging and treatment response evaluation. The integration of multimodal imaging data through radiomics not only enhances diagnostic precision but also supports longitudinal monitoring of fibrosis progression. This dynamic tracking capability allows clinicians to evaluate therapeutic responses in real time and adapt treatment strategies accordingly. For example, in patients receiving antifibrotic therapy, changes detected through multimodal radiomics can serve as early indicators of treatment efficacy or failure, helping to guide personalized and timely clinical interventions [36, 37]. Its ability to synthesize complex data from multiple modalities will likely play a pivotal role in early diagnosis, treatment response evaluation and long-term management of diabetic kidney fibrosis (Table 1).

**Table 1:** Advanced imaging modalities for non-invasive assessment of renal fibrosis.

Imaging technology	Main applications	Research progress and findings	References
MRI	Renal cortical thickness, texture analysis, dynamic contrast-enhanced scanning	Renal cortex thinning correlates with DKD progression; texture features (e.g. GLCM, GLRLM) aid early detection; DCE-MRI measures GFR and vascular enhancement; ADC values inversely relate to renal fibrosis severity	[5–8]
MRI DWI	Non-invasive evaluation of renal fibrosis	ADC values are positively correlated with renal function markers (e.g. eGFR); lower ADC values indicate more severe fibrosis	[7, 8]
MRE	Measurement of renal tissue elasticity to assess fibrosis	Medullary stiffness is significantly correlated with histological fibrosis; MRE can detect renal fibrosis and track disease progression	[10, 11]
SWE	Non-invasive evaluation of renal tissue elasticity	Renal fibrosis regions show stiffer tissue; YM predicts progression, while SWE with radiomics enables precise fibrosis quantification	[12–18]
CT	Observation of renal morphological changes and blood perfusion	Renal volume reduction correlates with fibrosis progression; contrast-enhanced CT assesses renal enhancement and blood perfusion levels	[20–25]
Multimodal radiomics	Integration of multiple imaging techniques to improve fibrosis evaluation accuracy	Multimodal radiomics combining MRI, CT and SWE can comprehensively capture the microscopic changes in fibrosis; multimodal radiomics models can predict the severity and progression of renal fibrosis	[27–32]
AI and ML	Automation and efficiency enhancement	Radiomics models combined with DL can automatically extract fibrosis-related features, reducing human error and increasing the automation and accuracy of fibrosis evaluation	[38–43]

## AI- AND ML-BASED FIBROSIS EVALUATION

Radiomics, especially when combined with AI and ML, holds great promise for the non-invasive diagnosis and monitoring of DKD [38]. However, one of the main challenges in AI models, particularly in radiomics, is the lack of interpretability. Many AI models, especially DL models, are often seen as “black boxes,” where the decision-making process is not transparent. This lack of transparency is a significant barrier to the adoption of AI in clinical settings, as clinicians need to understand how a model arrives at its conclusions in order to trust and act on its predictions [39]. While emerging techniques such as Explainable AI (XAI) aim to provide human-understandable explanations of model predictions, their application in radiomics for DKD is still in its early stages. Techniques like SHAP (Shapley Additive Explanations) and LIME (Local Interpretable Model-Agnostic Explanations) can help explain the contributions of different features to a model’s decision, thus improving transparency [40]. Currently, there are limited studies specifically addressing interpretability in the context of radiomics models for DKD. Nevertheless, the potential of XAI to make AI-driven insights more accessible and understandable to clinicians is substantial. In the future, XAI could play a critical role in making AI-based models in radiomics not only more accurate but also more interpretable and clinically relevant.

Another key challenge in applying AI models in radiomics is the large amount of annotated data required to train robust models. Obtaining large, well-annotated datasets can be difficult, especially in clinical settings where data are often scarce. This issue is particularly prominent in rare diseases such as DKD, where large patient cohorts are not always available for training purposes. To overcome this limitation, we propose leveraging transfer learning and few-shot learning techniques. Transfer learning enables models pre-trained on large public datasets to be fine-tuned on smaller, domain-specific datasets. This approach has shown success in other fields, such as radiology and oncology [41], where public image databases have been used to train models that are later adapted to specific diseases, reducing the need for large datasets in niche areas like DKD [42].

Despite the growing body of research, the lack of DKD-specific radiomics validation remains a significant gap. For radiomics-based diagnostic tools to be fully integrated into clinical practice, substantial progress must be made in large-scale validation studies, real-world evidence (RWE) and regulatory pathways. Recent large-cohort studies and multicenter trials are pivotal in establishing the robustness and clinical utility of radiomics-based models in DKD. A notable example is a recent study by Calvaruso *et al*. that utilized CT-based radiomics to predict the decline in renal function in patients with autosomal dominant polycystic kidney disease, achieving an AUC of 0.78. Such studies are essential to ensure that radiomics models can be generalized across different populations and settings [43]. These validation efforts are crucial as they help transition radiomics from proof-of-concept studies to widespread clinical use.

Incorporating RWE is another key step toward the clinical integration of radiomics. In oncology, real-world data have been used to support the performance and generalization of imaging biomarkers, demonstrating their effectiveness outside controlled experimental environments [44]. For DKD, leveraging RWE to validate radiomics models could allow for more accurate risk stratification and personalized treatment strategies. However, challenges remain in terms of data integration, harmonization and variability across clinical settings. As seen in oncology, RWE can play an instrumental role in bridging the gap between clinical trials and routine clinical practice. Regulatory pathways also represent a critical hurdle for the clinical adoption of radiomics-based diagnostic tools. Many radiomics models are classified as “Software as a Medical Device” (SaMD) by regulatory agencies such as the US Food and Drug Administration (FDA) and the European Medicines Agency (EMA). These models must undergo rigorous evaluation for safety, effectiveness and usability, as well as compliance with data privacy and fairness regulations. Currently, no radiomics-based diagnostic tools have been FDA-cleared or EMA-approved specifically for use in DKD. This underscores the need for further multicenter prospective trials, real-world deployment studies and engagement with regulatory agencies to facilitate the approval process and clinical integration of these tools.

## CONCLUSIONS AND FUTURE PERSPECTIVES

Radiomics has emerged as a powerful non-invasive and quantitative imaging technique, offering transformative potential in the research and clinical management of DKD. Compared with conventional diagnostic approaches, radiomics provides superior sensitivity in capturing subtle structural abnormalities, such as early glomerulosclerosis or tubulointerstitial fibrosis, that are often undetectable by traditional biomarkers or routine imaging. By quantifying these microscopic alterations, radiomics enhances disease staging accuracy and prognostic assessment, offering clinicians a more detailed and data-driven basis for clinical decision-making. In early disease stages, radiomics can detect microvascular changes that signal an elevated risk of progression, enabling timely interventions to delay renal function decline. Moreover, by comparing radiomic features before and after therapy, clinicians can objectively assess treatment efficacy and tailor therapeutic strategies accordingly. This quantitative paradigm not only improves diagnostic precision but also contributes novel insights into the underlying pathophysiology of DKD.

In assessing renal fibrosis in DKD, a variety of diagnostic modalities and radiomics models have been explored. Each method offers unique advantages, but also presents certain limitations that can impact their clinical applicability and diagnostic accuracy. To provide a clearer understanding of the comparative diagnostic performance, we have summarized key metrics—such as AUC, sensitivity and specificity—for different imaging modalities (MRI, CT, US elastography) and radiomics models in Table 2. This table highlights the strengths and weaknesses of each approach, offering valuable insights for clinicians when selecting the most appropriate method for evaluating renal fibrosis in DKD patients. It is important to note that while the values presented in the table, such as AUC, sensitivity and specificity, offer useful comparisons, they may not be directly comparable across all methods. Additionally, the included models were not always applied to the same patient cohorts, which may have influenced their diagnostic accuracy. By comparing these performance indicators, we aim to facilitate informed decision-making and guide future research in optimizing diagnostic strategies for this critical aspect of DKD management.

**Table 2:** Comparative diagnostic performance of different modalities for renal fibrosis in DKD.

Modality	AUC	Sensitivity %	Specificity %	Strengths	Limitations
MRI (radiomics model) [45]	0.98	92.3	86.7	Highresolution, detailed anatomical images	High cost, limited availability, long scan time
CT (radiomics model) [46]	0.79	70.9	73.2	Non-invasive, widely available, high spatial resolution	Radiation exposure, contrast agent requirement
SWE [33]	0.83			Cost-effective, portable, non-invasive	Operator dependency, limited sensitivity for early fibrosis

Additionally, coupling radiomics with multi-omics datasets, including genomics, transcriptomics, proteomics and metabolomics, promises to deepen mechanistic understanding and refine patient stratification. In recent years, there has been increasing interest in integrating radiomics with genomics, proteomics and metabolomics to enhance disease diagnosis, prognosis and treatment. In oncology, successful examples of radiogenomics have demonstrated the power of combining imaging features with genomic data, improving diagnostic accuracy and therapy prediction. For instance, a study by He *et al*. reviewed how integrating radiomic features from MRI with genomic mutations or gene expression data significantly enhanced tumor response prediction, achieving better accuracy than either modality alone [47]. Another study by Waqas *et al*. reviewed DL methods for the integration of multimodal data (imaging, pathology images, genomics, proteomics, metabolomics and clinical data) in oncology [48]. Although these studies primarily focus on cancer, the same principle of combining imaging and molecular data can be applied to DKD, where integrating imaging features with molecular data could provide a more comprehensive view of renal fibrosis progression. However, despite the promising potential, the integration of radiomics with genomics, proteomics or metabolomics in DKD remains largely unexplored. Currently, there are very few studies specifically targeting DKD renal fibrosis using radiomics in combination with other omics data. To fill this gap, we propose a conceptual framework for integrating radiomics with multi-omics data (genomics, proteomics and metabolomics) in DKD. This integration could improve patient stratification, provide more accurate diagnostic predictions and ultimately enable personalized treatment strategies.

**Figure 2:**
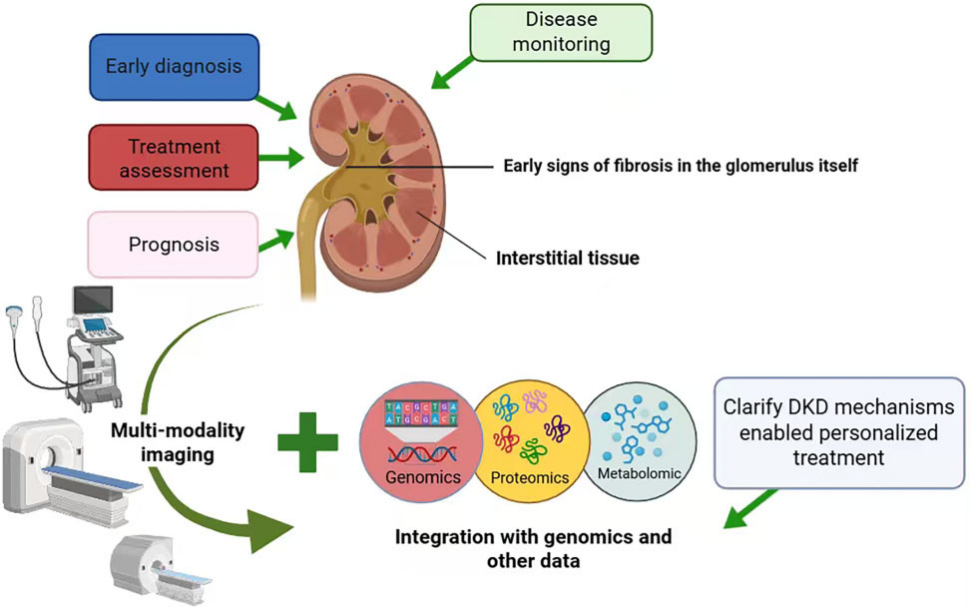
Radiomics, as a noninvasive and quantitative imaging analysis tool, offers great potential for DKD research and clinical applications. Multimodality imaging enables early diagnosis, prognosis, treatment response assessment and dynamic disease monitoring in DKD. Early fibrotic changes in glomeruli and interstitial tissue can be detected through radiological features. Integration with omics data—including genomics, proteomics and metabolomics—improves the understanding of DKD pathogenesis and supports personalized therapeutic strategies, enhancing clinical decision-making and precision medicine applications.（Figure created with BioRender.com）

Radiomics holds vast potential in advancing personalized medicine for DKD. Through individualized feature analysis, it enables patient-specific diagnostic profiling, risk stratification and optimization of therapeutic regimens. By predicting treatment responses based on imaging phenotypes and molecular data, radiomics can guide the selection of targeted therapies, improve outcomes and minimize adverse effects. Furthermore, it provides a framework for identifying high-risk individuals who may benefit from more intensive management. As technological advancements continue and large-scale clinical data accumulate, radiomics is poised to become an indispensable component of DKD management. Its development will foster interdisciplinary collaboration across medical imaging, nephrology, bioinformatics and systems biology—ultimately contributing to more precise, proactive and personalized care for patients with DKD (Fig. 2).

Despite the promising potential of radiomics in diagnosing and monitoring DKD, several critical gaps remain in the current research landscape. One of the primary limitations is the small sample sizes and single-center designs that characterize many studies [33, 49]. These limitations may hinder the generalizability of findings to diverse patient populations seen in clinical practice. Moreover, most studies are cross-sectional or retrospective in nature, with a lack of long-term follow-up data, making it challenging to assess the true predictive value of radiomics models in relation to disease progression and treatment outcomes. Additionally, the majority of existing studies have not validated radiomics models across different clinical settings, imaging devices and patient populations. This issue is particularly prominent in DKD, where the mechanisms of renal fibrosis and disease progression can vary significantly between individuals.

To address these gaps, future research should focus on conducting prospective clinical trials to evaluate the ability of radiomics models, when combined with clinical biomarkers and other omics data, to predict disease progression and treatment outcomes in DKD patients. These trials should include larger cohorts with long-term follow-up to provide robust data on the predictive value of radiomics in clinical practice and its impact on patient management. By assessing the performance of radiomics models over time, these studies will enable a clearer understanding of how these tools can be integrated into routine clinical workflows.

In addition, large-scale multicenter studies are essential to ensure the generalizability and external validity of radiomics models. These studies should be designed to test the performance of radiomics models in diverse patient populations, including those with varying stages of DKD, and across different imaging devices and protocols. Multicenter validation is crucial to confirming the robustness and reliability of radiomics models for clinical use in various settings, making them more adaptable for widespread application.

Given the high costs associated with implementing advanced radiomics models and AI technologies, future studies should also include cost–benefit analyses to assess the economic feasibility of integrating these models into routine clinical practice. Evaluating both the clinical and economic impacts of radiomics will provide a clearer understanding of its value in improving diagnostic accuracy and patient outcomes, as well as its sustainability in real-world clinical settings.

By addressing these gaps in research, we believe that radiomics has the potential to significantly enhance the diagnosis, monitoring and management of DKD. However, further studies are needed to validate these models across larger, more diverse patient populations and to assess their economic viability in real-world clinical environments.

## Data Availability

The data underlying this manuscript are available in the article and in its online supplementary material.
